# mRNA-Based Approaches to Treating Liver Diseases

**DOI:** 10.3390/cells11203328

**Published:** 2022-10-21

**Authors:** Maximiliano L. Cacicedo, María José Limeres, Stephan Gehring

**Affiliations:** Children’s Hospital, University Medical Center Mainz of the Johannes-Gutenberg University, Langenbeckstr. 1, 55131 Mainz, Germany

**Keywords:** therapeutic mRNA constructs, lipid nanoparticles, cirrhosis, inherited metabolic diseases, hepatocellular carcinoma

## Abstract

Diseases that affect the liver account for approximately 2 million deaths worldwide each year. The increasing prevalence of these diseases and the limited efficacy of current treatments are expected to stimulate substantial growth in the global market for therapeutics that target the liver. Currently, liver transplantation is the only curative option available for many liver diseases. Gene therapy represents a valuable approach to treatment. The liver plays a central role in a myriad of essential metabolic functions, making it an attractive organ for gene therapy; hepatocytes comprise the most relevant target. To date, viral vectors constitute the preferred approach to targeting hepatocytes with genes of therapeutic interest. Alternatively, mRNA-based therapy offers a number of comparative advantages. Clinical and preclinical studies undertaken to treat inherited metabolic diseases affecting the liver, cirrhosis and fibrosis, hepatocellular carcinoma, hepatitis B, and cytomegalovirus using lipid nanoparticle-encapsulated mRNAs that encode the therapeutic or antigenic protein of interest are discussed.

## 1. Introduction

The liver is the largest internal organ of the body. It maintains metabolic homeostasis, performing an enormous number of vital functions that include the following: (1) blood filtration, purification, and detoxification; (2) metabolism of fats, proteins, and carbohydrates; (3) storage of glycogen, vitamins, and minerals; (4) bile production and excretion; (5) excretion of bilirubin, cholesterol, hormones, and drugs; (6) enzyme activation; (7) protein synthesis, and amino acid metabolism. Diseases that affect the liver account for approximately 2 million deaths annually worldwide; most consist of viral hepatitis (A, B, and C), cancer due to hepatitis and hepatocellular carcinoma (HCC), alcoholic liver disease, fatty liver and cirrhosis, and hereditary diseases [1,2]. Cirrhosis of the liver is the eleventh most common cause of death globally, while liver cancer due to viral hepatitis and HCC is the sixteenth leading cause [3,4]. According to the American Cancer Society, an estimated 41,260 new cases will occur, and 30,520 patients will die from primary liver cancer and cholangiocarcinoma (bile duct cancer) in the United States in the year 2022. Risk factors for HCC include chronic viral hepatitis, alcohol addiction, metabolic liver disease (particularly nonalcoholic fatty liver disease), and exposure to dietary toxins such as aflatoxins and aristolochic acid. Purportedly, all of these contributing factors are preventable [2].

The increasing prevalence of liver diseases and the limited efficacy of treatments currently available are expected to propel substantial growth in the global market for therapeutics that target the liver [5]. The principal market consists of the following: anti-viral drugs, chemotherapeutics, vaccines, immunoglobulins, corticosteroids, anti-rejection and immunosuppressants, and drugs for targeted therapy; the antiviral drug segment comprised the largest market share in 2019. Currently, organ transplantation is the only curative option available for many liver-associated diseases [6]. Indeed, inherited metabolic liver diseases, which account for 10–15% of cases (22–65% mortality rate) of acute liver failure in children, are the second most common indication for pediatric liver transplantation [7]. Gene therapy, enabled by the rapid expansion of genomic data, represents a valuable alternative approach to treating many of these diseases.

The liver is an attractive organ for gene therapy since it plays a central role in a myriad of essential metabolic functions. It contains 10–15% of the total blood volume in the body, making it ideal for the production and secretion of proteins into circulation. Hepatocytes, which comprise 70–80% of the total liver cell population, constitute the most relevant hepatic target cells for gene therapy [8]. Notably, hepatocytes play a key pathogenic role in many hepatic disorders due to their broad range of functions [5]. To date, viral vectors, for example, attenuated recombinant viruses such as an adeno-associated virus (AAV) or lentivirus, represent the preferred approach to targeting hepatocytes with genes of therapeutic interest [9]. A potential disadvantage of using viral vectors (e.g., AAV) is pre-existing immunity, especially the presence of circulating neutralizing antibodies [10,11]. In the case of AAV, pre-existing immunity can be circumvented by selecting a variant of the vector that is not widely circulated in the human population. It is equally important to note the infrequent integration of recombinant AAV into the genome and the subsequent occurrence of genotoxicity in animal models [10,11].

DNA-based therapeutic approaches require efficient cytoplasmic delivery and nuclear entry of the DNA to ensure transcription, mRNA production, and the synthesis of therapeutic proteins that follow. In this regard, nuclear delivery is a major obstacle for DNA therapeutics since the majority of terminally differentiated cells, e.g., mature hepatocytes, are post-mitotic and do not undergo frequent cell division [12]. Another limitation, the “dilution effect,” occurs when therapy becomes less effective with time due to the growth and regeneration of a younger cell population [13].

mRNA-based therapy offers a number of advantages over DNA-based therapeutic approaches. mRNA expression does not require entrance into the cell nucleus for expression to occur, lowering the barrier for functional delivery [12,14]. Moreover, mRNA is incapable of integrating into the genome, eliminating the potential risk of insertional mutagenesis; rather, mRNA is degraded upon protein synthesis, thus repeated inoculation is required [15]. In contrast to traditional gene therapy, the transient nature of mRNA expression permits precisely controlled dosing dependent upon the clinical requirements. Less durable expression and the requirement for repeated dosing to sustain therapeutic efficacy, on the other hand, could be a limiting factor depending upon the target. A final advantage of mRNA-based therapeutic approaches, compared to viral vector-mediated gene transfer, is that in vitro-transcribed mRNA can be produced rapidly in large quantities at a relatively lower cost by cell-free processes [15,16].

## 2. Therapeutic, mRNA Constructs

The potential efficacy of in vitro-transcribed mRNA used to treat liver diseases is based upon their ability to encode proteins that replace impaired hepatic functions using the translational machinery of the target cells, i.e., hepatocytes [17]. Therapeutic mRNA offers a distinct advantage over protein replacement or enzyme replacement therapy used to restore the functional proteins that are otherwise deficient or abnormal: mRNA delivered and expressed intracellularly allows post-translational modifications of the encoded protein by the host cells [18]. The utility of RNA expression constructs to stimulate protein production was first described in 1990 in mice injected intramuscularly (i.m.) [19]. For decades since then, however, the use of RNA for therapy was considered impractical due to the following: (1) inherent instability and vulnerability to nuclease digestion, (2) tendency to induce inflammation and strong innate immune responses, and (3) inability to readily cross the cell membrane and enter the cytoplasm [15]. Recent technical advances that circumvent these obstacles have optimized mRNA molecules and maximized their therapeutic potential by engineering them to display low immunogenicity, prolonged stability, and potent translation efficiency [15,16].

**Figure 1:**
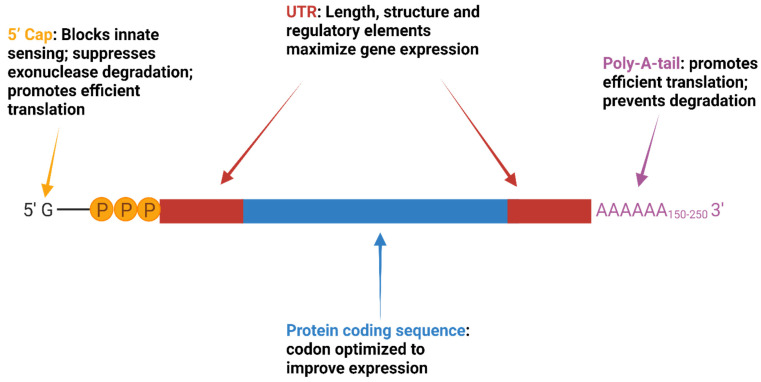
mRNA vaccine/therapeutic protein construct.

Therapeutic mRNA constructs are small and simple. They encode the following: the protein of interest, flanked by 5′ and 3′ untranslated regions (UTRs); a 5′ cap structure consisting of 7-methylguanosine (m^7^G) connected by a triphosphate bridge to the first nucleotide of the 5′UTR, and a 3′-poly-(A) tail [20,21,22,23,24]. The elements of therapeutic mRNA constructs and their purported functions are illustrated in Figure 1.

Codon optimization, which involves a selection of the most abundant tRNA-related codons and nucleoside modification (e.g., substituting pseudouridine for uridine) diminishes Toll-like receptor (TLR) recognition, suppresses innate immune responses to mRNA, and enhances translation efficiency [25,26]. mRNA purity is crucial; small oligoribonucleotide and double-stranded RNA sequences generated during construct synthesis are recognized by pattern recognition receptors, e.g., TLR [27,28]. Removal of these impurities promotes translation and protein synthesis by suppressing innate immunity and the production of inflammatory cytokines [29]. Figure was created with biorender.com.

## 3. Delivery Vehicle

Although chemical modifications and sequence engineering improve the translation and shelf life of synthetic mRNA, mRNA alone is unsuitable for therapy [15]. The development of efficient delivery systems is key to advancing mRNA-based therapeutics. Cellular uptake and translocation are the biggest barriers to mRNA expression; the negative potential across the cell membrane is formidable. mRNA, which is prone to nuclease digestion, is too large and negatively charged to cross the cell membrane passively; relatively little is internalized and translated, and most is rapidly degraded [30]. Furthermore, mRNA injected directly into either animals or humans elicits severe inflammation and an innate immune response.

**Figure 2:**
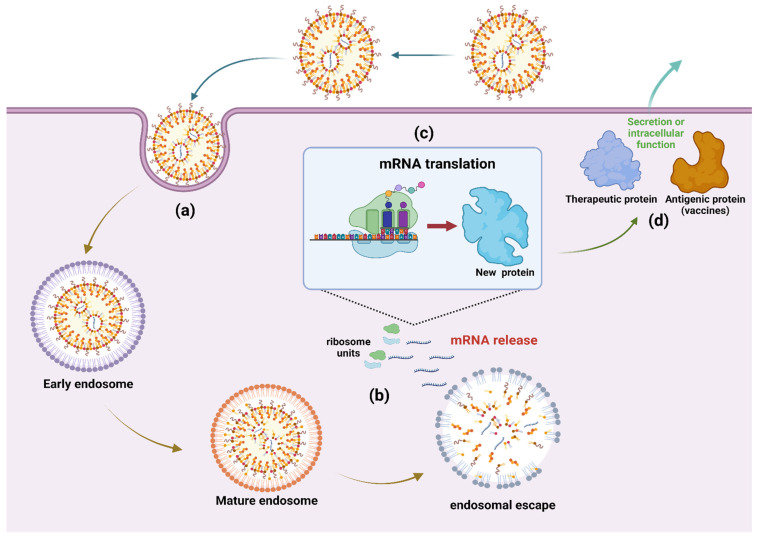
Cellular uptake and expression of LNP-encapsulated mRNA constructs. Constructs encoding antigenic/therapeutic proteins are encapsulated in LNP to prevent degradation and to promote cellular uptake: (**a**) Uptake of the mRNA-LNP complex is mediated by endocytosis; (**b**) mRNA constructs are released from the endosome into the cytosol where they are translated by ribosomes; (**c**) the antigenic/therapeutic proteins are produced; (**d**) The protein products are sequestered intracellularly, incorporated into cell membranes or secreted. Figure was created with biorender.com (accessed on 19 August 2022).

The incorporation of synthetic mRNA into a delivery vehicle significantly alters its inflammatory profile and therapeutic potential [31,32]. The principal functions of the vehicle are to protect the message from extracellular nuclease digestion and to facilitate uptake by host cells, which occurs primarily by endocytosis (Figure 2) [33]. Once internalized, the delivery vehicle must promote escape from endosomes and release its contents into the cytosol for translation. While internalization is a relatively simple process, the endosomal membrane represents a significant obstacle to the release and subsequent expression of intact mRNA [34].

Non-viral vectors have emerged recently as highly efficient vehicles for the transfer of genetic information, i.e., therapeutic mRNA molecules. The following two classes exhibit considerable efficacy: (1) cationic polymers (polycations), which combine with nucleic acids through electrostatic interactions to form polyplexes; (2) lipid nanoparticles (LNPs) composed of amphiphilic lipids that, when dispersed in an aqueous environment, spontaneously form spherical structures with a hydrophilic interior upon interaction with negatively charged molecules such as mRNA [31,35]. LNPs are suitable carriers for nucleic acid delivery, they exhibit excellent biocompatibility, biodegradability, low toxicity and immunogenicity, structural flexibility, and ease of large-scale preparation. LNPs are by far the most common non-viral gene carriers used to date [35].

**Figure 3:**
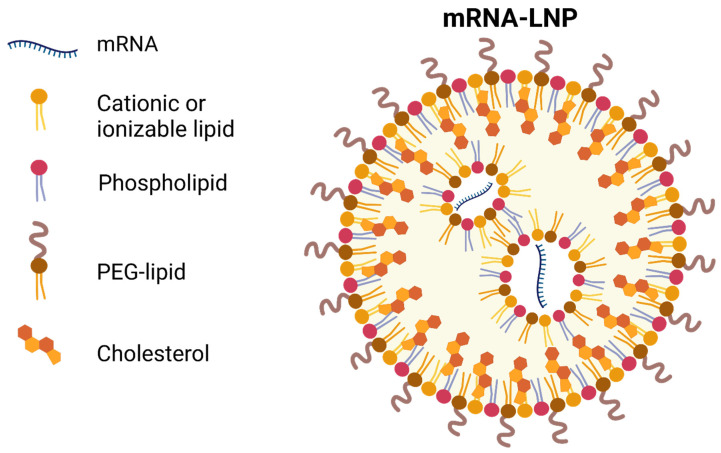
Lipid nanoparticle-encapsulated mRNA construct. LNP-encapsulated mRNA constructs are composed of: (1) polyanionic mRNA bound by an ionizable lipid, (2) a zwitterionic phospholipid that helps package nucleic acids and stabilize LNPs, (3) cholesterol, which stabilizes the LNP lipid bilayer and promotes fusion with the cell membrane, and (4) lipid-anchored PEG, which reduces non-specific protein absorption, diminishes LNP aggregation and improves colloidal stability. Figure was created with biorender.com (accessed on 19 August 2022).

Cell membranes, composed primarily of a lipid bilayer of zwitterionic and negatively charged phospholipids, create a challenging barrier for highly negatively charged mRNA molecules [35,36]. Recent studies have focused on the development of novel, biocompatible lipid formulations that facilitate cellular uptake, endosomal release, and mRNA expression [34,37]. Typically, LNPs are synthesized by mixing mRNA in an acidic aqueous phase with an ethanol phase that contains a precise molar ratio of the following: (1) an ionizable lipid, (2) a zwitterionic phospholipid, (3) cholesterol, and (4) lipid-anchored polyethylene glycol (PEG) (Figure 3) [35,38]. LNPs usually contain only a few (1–10) mRNA copies, which are bound by the ionizable lipid and located in the nanoparticle core [39]. 

LNPs in the bloodstream exhibit a net neutral surface charge but become positively charged in acidified endosomes once internalized, leading to mRNA unfolding and release into the cytoplasm [40]. The ionizable lipid is the principal factor determining LNP efficacy [38]. Specific LNPs formulations are often proprietary, but a number of proprietary ionizable lipids incorporated into LNPs were evaluated to determine which maximized the expression of encapsulated mRNA [41]. LNP formulations are often designed based on the target tissue, application, and route of administration. LNPs that incorporate MC3 ionizable lipids, for example, successfully deliver mRNA to hepatocytes after i.v. administration [42,43]. Notably, efficient delivery and translation of mRNA in the liver have also been reported after i.m. administration [44]. The inclusion of biodegradable lipids with short half-lives improves LNP safety and tolerability, which are key factors in the performance of any new therapeutic construct. Rapid metabolism or excretion correlates with a reduction in inflammation at the injection site and the adverse consequences that attend to accumulation in the tissues [32].

Therapeutic mRNA formulated in LNPs is most often inoculated intravenous (i.v.) or i.m. and will likely require repeated administration in order to sustain therapeutic protein levels [45]. The dosing frequency will depend upon the protein half-life, its activity, and the turnover rate of the target cell. The average half-life of protein production following mRNA transfection in vivo ranges from 7 to 30 h, dependent upon the route of administration [46].

## 4. Clinical and Preclinical Applications

To date, clinical efforts devoted to treating diseases using LNP-formulated RNA constructs have focused largely on the development of prophylactic or therapeutic vaccines for infectious and malignant diseases. mRNA-based therapeutic approaches to treating a variety of liver diseases, however, are currently under investigation.

### 4.1. Inherited Metabolic Diseases

**Figure 4:**
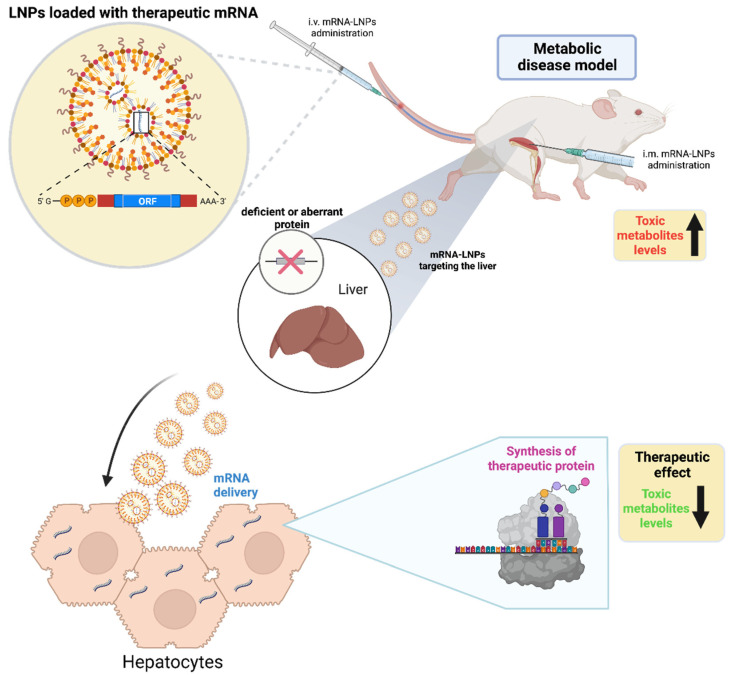
mRNA-based approach for enzyme replacement therapy. In general, mouse models for metabolic disorders from the liver constitute enzyme-deficient mice characterized by the accumulation of toxic serum concentrations of metabolites. mRNA-LNPs inoculated i.m. or i.v. are transported via the bloodstream to the liver where the message is translated, and the protein is synthesized by hepatocytes. Newly produced protein replaces the deficient or aberrant protein associated to the disease resulting in the rescue of metabolic function and fast reduction of toxic metabolite levels, consequently generating a therapeutic effect. Figure was created with biorender.com (accessed on 19 August 2022).

Inherited metabolic disorders are important causes of morbidity and mortality in children [6]. The liver is the source of many of these disorders, which occur in approximately 1:800 live births. They are typically caused by an autosomal recessive mutation in a single gene [7]. Inherited metabolic diseases account for 10–15% of cases (22–65% mortality rate) in pediatric patients with acute liver failure [7]. Metabolic liver diseases are the second most common indication for liver transplantation in children [6,47]. Next-generation sequencing (NGS) technology has enabled scientists to identify the genetic basis for many of these diseases [48,49]. NGS methods have led to significant reductions in the time and cost required to sequence entire genomes [50]. While useful for diagnostic purposes, this information has yet to be translated into pharmaceutical interventions that address the genetic defects that underlie the diseases. Most interventions, which are still in the preclinical stages of development, involve protein or enzyme replacement therapy to replace deficient or aberrant proteins. For most of these diseases, protein replacement is not an option; the only curative option is transplantation. Limitation in enzyme replacement includes the following: variability in a patient’s response, production of neutralizing antibodies, infusion reactions, and glycosylation pattern that affects the immunogenicity and/or function of the recombinant protein [17]. mRNA-based therapy offers an alternate approach. A major advantage associated with mRNA-based therapy is that the protein product is synthesized and modified by natural intracellular machinery, ensuring the following proper: folding, intracellular location, and post-translational processing (Figure 4).

Hepatocytes are the most relevant hepatic target cell type for gene therapy. They are highly polarized with a sinusoidal (basolateral) membrane positioned towards the blood circulation and an apical membrane towards bile canaliculi. The sinusoidal membrane expresses surface receptors important for LNP recognition; apolipoprotein E and the asialoglycoprotein receptor are the most important [51].

The efficacy of mRNA-based protein replacement therapy utilizing systemic delivery of liver-targeting LNPs has been demonstrated in a number of animal models [12,17]. The following rare genetic disorders that affect the liver have been treated using mRNA in preclinical studies; a number have entered clinical trials.

#### 4.1.1. Hereditary Tyrosinemia Type 1 (HT1)

HT1 is an inborn error in amino acid metabolism caused by a deficiency in functional fumarylacetoacetate hydrolase (FAH), which results in the accumulation of toxic and carcinogenic metabolites [52,53,54]. HT1 patients are at an increased risk of developing neurologic symptoms, renal failure, and early-onset HCC. The standard of patient care consists of a strict life-long diet low in tyrosine and phenylalanine that is supplemented with nitisinone, 2-(2-nitro-4-trifluoromethyl benzoyl) cyclohexane-1, 3-dione (NTBC), taken orally twice daily [53].

Several point mutations affect the *FAH* gene in patients suffering from HT1 [55]. Recently, we reported that NTBC-deprived, *Fah*-deficient mice injected with *FAH* mRNA-LNPs exhibited prolonged FAH synthesis in the liver, sustained body weight, and drastically reduced toxic concentrations of tyrosine and succinylacetone in the serum [44]. Cheng et al., reported similar findings [56]. *Fah*-deficient mice administered *FAH* mRNA encapsulated in dendrimer-based LNPs showed no signs of disease, weight loss, or liver complications. Taken together, these findings support the potential use of an mRNA-based therapeutic approach to treat HT1.

#### 4.1.2. Phenylketonuria (PKU)

PKU is an inborn error in metabolism caused by a deficiency in functional phenylalanine hydroxylase (PAH), leading to the accumulation of phenylalanine (Phe) in the blood and organs of patients [57,58]. Untreated patients suffer severe neurological impairment. A diet restricted in Phe is fundamental to disease management. Often, however, dietary restrictions are not entirely effective. Currently, there are the following two approved drugs on the market used to treat PKU: sapropterin dihydrochloride and pegylated phenylalanine ammonia lyase. Neither is effective in treating a majority of PKU patients. Alternate therapeutic approaches are needed.

Repeated i.v. injection of mouse *Pah* (*MmPah*) mRNA formulated in LNPs into a PKU (*Pah^enu2^*) mouse model resulted in therapeutic PAH protein production in the liver, decreased Phe concentrations in the serum, liver, and brain, and reversed disease progression [59]. Perez-Garcia and coworkers reported similar findings [60]. These results suggest that LNP-formulated *Pah* mRNA could provide an alternate treatment option for PKU patients that circumvents life-long adherence to a Phe-restricted diet. In this regard, the ModernaTx, Inc. (Cambridge, MA, USA) website (www.modernatx.com/research/product-pipeline, accessed on 1 August 2022) lists *PAH Phenylketonuria (PKU)* mRNA-3283 in its pipeline for product development.

#### 4.1.3. Methylmalonic Acidemia (MMA)

Isolated MMA is an organic acidemia with significant rates of morbidity and mortality, and no approved therapies that address the underlying defect [61]. It is an autosomal recessive disorder characterized by the impaired metabolism of propionate derived from certain proteins and fats, and the marked elevation of methylmalonic acid, in body fluids and tissues [62]. A deficiency in the mitochondrial enzyme, methylmalonyl-coenzyme A (CoA) mutase (MUT), is the most frequent cause. Disease management is limited to stringent dietary restrictions. Liver transplantation leads to a significant reduction in circulating methylmalonic acid indicating that the liver is a major metabolic organ for the disorder. LNP-encapsulated *MUT* mRNA administered systemically offers an alternate approach to restoring the synthesis of functional MUT enzymes in the liver. Indeed, hypomorphic Mut^−/−^; Tg^INS-CBA-G715V^ mice inoculated repeatedly i.v. with LNP-encapsulated *MUT* mRNA exhibited a reduction in plasma MMA concentrations and an increased rate of survival [63,64]. Importantly, safety studies found no changes in liver function tests, inflammatory cytokine production, or the synthesis of anti-MMA antibodies. A phase I/II clinical trial is currently underway to determine the safety, pharmacokinetics, and pharmacodynamics of LNP-encapsulated human *MUT* mRNA (mRNA-3705) administered to patients with isolated methylmalonic acidemia (Moderna; clinicaltrials.gov (accessed on 1 August 2022) Identifier: NCT04899310).

#### 4.1.4. Propionic Acidemia (PA)

Propionyl-CoA carboxylase (PCC), which catalyzes the carboxylation of propionyl-CoA to methylmalonyl-CoA, is a hetero-dodecamer encoded by the *PCCA* and *PCCB* genes. PA is a pediatric disorder caused by a mitochondrial deficiency in PCC, impairing propionate metabolism and leading to the accumulation of toxic metabolites, i.e., 2-methylcitrate, 3-hydroxypropionate, and propionyl carnitine [65]. 

Symptoms commonly present during the first weeks of life include vomiting, lethargy, hypotonia, dehydration, and failure to thrive. LNP-encapsulated *PCCA* mRNA and *PCCB* mRNA injected i.v. resulted in the synthesis of therapeutic levels of PCCA and PCCB in the livers of a hypomorphic disease model (Pcca^−/−^[p.A138T]) in mice [66]. Repeated dosing of *PCCA* and *PCCB* mRNAs encapsulated in LNPs over the course of a 6-month period was well-tolerated, toxic metabolite levels in plasma were reduced but not quite normalized, liver transaminases were normal, and adverse reactions were nonattending. The results of this study support the ongoing Phase 1/2 study designed to evaluate the safety and pharmacodynamic activity of mRNA-3927 (LNP-encapsulated *PCCA* and *PCCB* mRNAs) administered to PA patients 1 year of age or older (clinicaltrials.gov Identifier: NCT041591030).

#### 4.1.5. Glycogen Storage Disease Type 1a (GSD1a)

GSD1a is a metabolic disorder caused by an autosomal recessive mutation in the gene that encodes the catalytic subunit of glucose-6-phosphatase (G6Pase), which hydrolyzes glucose-6-phosphate to yield free glucose. The liver is the first organ affected since it is the principal site of gluconeogenesis. GSD1a symptoms include hypoglycemia, hypertriglyceridemia, anemia, renal disease, and a life-long risk of HCC; currently, there are no curative treatment options available [67].

In a recent report, a liver-specific G6pc knockout mouse (L.G6pc^−/−^) was inoculated repeatedly i.v. with h*G6PC*-a mRNA encapsulated in LNP [68]. Treated mice exhibited a vast improvement in fasting glycemia and a significant reduction in GSD1a biomarkers (i.e., glycogen, G6P, and triglycerides). The serum cytokine levels (i.e., IFN-ɣ, IL-1β, TNFα, and IL-6) were comparable in treated and control animals. Moreover, treatment did not elicit an anti-G6Pase response, liver injury, change in body weight, or distress. These findings provide further support for studying the potential efficacy of LNP-encapsulated mRNA used to treat inherited metabolic disorders. A clinical trial was undertaken to determine the safety and tolerability, and to characterize the pharmacokinetic and pharmacodynamic response to a single dose of h*G6PC*-a mRNA encapsulated in LNP (mRNA-3745) injected i.v. into patients suffering from GSD1a is currently ongoing (clinicaltrials.gov ID NCT0595727).

#### 4.1.6. Ornithine Transcarbamylase (OTC) Deficiency

OTC catalyzes the reaction between carbamoyl phosphate and ornithine to form citrulline and phosphate [69]. It is a key enzyme in the urea cycle found in the liver that helps to eliminate ammonia. High ammonia levels can cause neuropsychiatric symptoms that range from mild to severe. Available treatments, i.e., a protein-restricted diet and ammonia scavengers, do not deal with the underlying cause. Liver transplantation is the only known cure.

Prieve et al., reported that a hyperammonemic murine model of OTC deficiency (*Otc^spf-ash^*) treated with NP-encapsulated h*OTC* mRNA (ARCT-810) exhibited normalization of plasma ammonia and orotic acid levels, an increased rate of survival, and a good safety profile [70]. A Phase 1b clinical trial (clinicaltrials.gov Identifier: NCT04442347) dedicated to determining the safety, tolerability, and pharmacokinetics of a single dose of ARCT-810 administered to clinically stable OTC deficiency patients is currently in progress.

In addition to the inherited metabolic diseases detailed above, preclinical animal studies have been conducted using mRNA-based therapeutic approaches to treat a number of other rare monogenetic liver disorders (Table 1). Pharmacokinetic studies generally demonstrated the rapid onset of mRNA expression following a single dose of mRNA-LNP injected i.v. Functional protein was restored to therapeutic levels in the livers of relevant animal models within a relatively short period of time; this protein often persisted despite significant mRNA degradation. Therapeutic protein levels were transient; however, maintenance required repeated mRNA-LNP dosing. Repeated mRNA–LNP administration is required to sustain therapeutic protein levels and treat chronic metabolic diseases and carries a risk of toxicity [49]. In the studies reviewed herein, however, therapy was effective and well tolerated after both single and multiple doses. The concentrations of toxic metabolites were reduced, and animals dosed repeatedly appeared distress-free, retained stable body weights, and experienced long-term survival. Importantly, mRNA therapy was well tolerated. There was no histologic or enzymatic evidence of liver injury in mice inoculated repeatedly; plasma concentrations of liver biomarkers (aspartate aminotransferase, alanine aminotransferase, alkaline phosphatase, and creatine kinase) persisted or improved. Moreover, in contrast to typical immunological responses to traditional enzyme or protein replacement therapy, there was never evidence of therapeutic protein-specific antibody production after repeated mRNA-LNP administration. This suggests that improved immune tolerance is inherent in mRNA-encoded therapeutic proteins that are produced endogenously by mechanisms that ensure proper post-translational processing, modification, glycosylation, and folding. These findings are supported by the results of two studies that reported experiments conducted with wild-type non-human primates [71,72]. Animals inoculated with LNP-encapsulated mRNA exhibited a marked increase in the protein encoded by the mRNA construct but no evidence of liver injury or anti-protein production. Notably, though, information regarding the safety of administering mRNA-LNP for a long period is scarce.

### 4.2. Acquired Liver Injury

Acute and chronic (cirrhosis) liver injuries are frequently caused by virus infections (hepatitis A, B, and C), exposure to hepatotoxins, e.g., excessive alcohol consumption, certain medications such as acetaminophen, and nonalcoholic fatty liver disease [80,81]. Treatment of mice in a model of acute liver injury (550 mg/kg acetaminophen injected i.p.) with mRNA expressing HGF/EGF formulated in LNPs stimulated hepatocyte division, liver regeneration, and improved liver pathology evidenced by a rapid return to baseline liver enzyme (ALT) levels [82]. Similarly, injection with LNP-formulated *HGF/EGF* mRNA stimulated a sharp reversal in steatosis and accelerated the restoration of liver function in mice fed a choline-deficient diet in a model of nonalcoholic fatty liver disease.

Fibrosis resulting from persistent liver damage is associated with the following down-regulated expression of the master regulator of hepatocyte phenotype: hepatocyte nuclear factor 4α. LNP-formulated human *HNF4α* mRNA reduced liver fibrosis and cirrhosis in a mouse model created by injecting 10% carbon tetrachloride in olive oil twice weekly for 8 or 16 weeks, respectively [83].

### 4.3. Primary Liver Cancer

Primary liver cancer is the sixth most commonly diagnosed cancer and the third leading cause of cancer-related deaths worldwide [2,3]. Greater than 80% of primary liver cancers are hepatocellular carcinomas (HCCs); the majority of cases occur in patients with cirrhosis due to chronic hepatitis B virus (HBV) or hepatitis C virus (HCV) infections, excessive alcohol consumption, or nonalcoholic fatty liver disease. Indeed, chronic inflammation is a primary risk factor for many human malignancies, including those affecting the liver [84]. The course of curative treatment consisting of resection, percutaneous ablation, transarterial chemoembolization, radioembolization, or transplantation is primarily dependent upon tumor burden, location, and comorbidities [85]. Although the prognosis varies among treated patients, the rate of tumor recurrence is generally high [86]. Adjuvant therapy decreases the risk of recurrence and provides survival benefits after surgical HCC resection [87].

Currently, kinase inhibitors (i.e., sorafenib and lenvatinib) administered as single-drug therapies constitute the first-line systemic treatment for advanced HCC cases [88]. Sorafenib is very effective in the early stages but wanes as the disease progresses. Regorafenib and cabozantinib (additional kinase inhibitors), and ramucirumab (monoclonal anti-vascular endothelial growth factor receptor-2 [VEGFR-2] antagonist that inhibits tumor angiogenesis) are administered as second-line therapies. Anti-tumor immunity can be suppressed by tumor-specific mechanisms that involve pathways that are not targeted. Consequently, only a minority of patients achieve durable responses to current therapies.

#### 4.3.1. Checkpoint Inhibitors

Treatment with monoclonal antibodies that block immune regulatory checkpoint receptors, i.e., immune checkpoint inhibitors (CPIs) such as atezolizumab and bevacizumab, monoclonal anti-PDL1 and anti-VEGF antibodies, respectively, increases the overall survival rate of unresected cases [88]. Used alone or in combination, CPIs stimulate immune responses to malignant cells by interrupting the inhibitory interaction of effector T cells with antigen-presenting tumor cells. These immune CPIs are now approved by the U.S. Food and Drug Administration (FDA) to treat a variety of cancers, including liver tumors [89]. Most patients, however, do not demonstrate durable benefits from these therapies; many tumor types are unresponsive or minimally responsive [90]. Indeed, only a fraction of patients with responsive tumors achieve lasting remission [84].

#### 4.3.2. Vaccines

Therapeutic immunization offers an alternate approach to treating primary liver cancer. In fact, the initial interest in mRNA-based therapy focused on its potential use in cancer treatment. Cancer vaccines, intended for treatment rather than prophylaxis, are designed to target tumor-associated antigens expressed preferentially by malignant cells and, consequently, to stimulate cell-mediated immune responses capable of reducing the tumor burden. Exploration of mRNA to induce adaptive immune responses to cancer began in 1995 when Conry and coworkers reported that protective antitumor immunity could be induced in mice by intramuscular injection of mRNA encoding carcinoembryonic antigens [91]. Currently, more than one hundred clinical trials for mRNA vaccines are listed by the U.S. National Library of Medicine (clinicaltrials.gov) for a wide range of cancers, including primary liver cancer. Most trials are early, but some have progressed to phase 2.

Initial approaches to vaccine development focused on shared antigens that were expressed by most patients [84]. These antigens, self-antigens, tended to be tissue-restricted and abnormally expressed by cancer cells, making them moderately cancer-type specific. A variety of tumor-associated antigens (TAAs) have been used in efforts to develop the following therapeutic vaccines against non-viral cancers: NY-ESO-1, MAGE-A3, BAGE, CEA, AFP, XAGE-1B, survivin, P531, h-TERT, mesothelin, PRAME, MUC-1 [92]. In general, none of the vaccine strategies that target these TAAs have garnered much success in clinical trials. mRNA-based cancer vaccines specific for additional (novel) TAAs need to be evaluated. In this respect, aspartyl/asparaginyl β-hydroxylase (ASPH), a promising target, is overexpressed in a variety of malignant tumors including HCC, but negligibly in normal tissues [93,94].

Recently, BioNTech received FDA fast-track approval to evaluate its vaccine candidate, BNT111, in a Phase II clinical trial to treat patients with advanced melanoma (clinicaltrials.gov Identifier: NCT04526899). BNT111 is an LNP-formulated mRNA vaccine candidate that encodes a fixed set of four TAAs (NY-ESO-1, MAGE-A3, tyrosinase, and TPTE); ≥90% of melanomas in patients express at least one of these four antigens [95].

#### 4.3.3. Personalized RNA Mutanome Vaccines

Personalized mRNA vaccine constructs offer an alternative method to immunizing primary liver cancer patients [96,97,98]. Somatic mutations are important promoters of cancer development. Many mutations are unique, leading to a distinct set of mutations in each patient’s tumor (the mutanome), defined by comparing exome sequencing data obtained by NGS of healthy and tumor-derived tissues [98]. The evidence suggests that a significant subset of these tumor-specific mutations encode neo-epitopes recognized by autologous T cells [99]. It is generally believed that these neo-epitopes represent the primary targets of an effective immune response induced as a consequence of immune CPI therapy [100,101,102]. In fact, the mutational burden often correlates with, but is not the sole factor that determines, the sustained clinical benefit of CPI therapy [100,102,103].

While immune CPI therapy can improve the overall prognosis of some patients with advanced malignancies, pharmacologic disruption of these immune checkpoints can lead to a wide range of inflammatory toxicities, collectively referred to as immune-related adverse events (irAEs) and the response to ‘self’ proteins (CPI-associated autoimmune syndrome) [89,101,104,105]. The majority of irAEs from checkpoint blockade involve either barrier tissues (e.g., gastrointestinal mucosa or skin) or endocrine organs [89]. Methods are needed for targeting the immune response activated by CPI therapy to mutations contained in the tumor while reducing activation of immune responses to normal tissue (i.e., irAEs). Conceivably, neoantigen-based vaccines offer such an approach [106].

Given the flexibility and ease of manufacturing, mRNA sequences encoding multiple neo-epitopes can be incorporated into a single, polyneoepitope backbone that comprises the personalized vaccine construct. The safety and clinical feasibility of this approach were demonstrated in a first-in-human trial undertaken to treat thirteen patients with metastatic melanoma (Clinical Identifier: NCT02035956) [98]. Each patient, immunized with a vaccine that encoded ten neo-epitopes unique to his/her tumor, exhibited CD4^+^ and CD8^+^T cell responses to selected epitopes. Antitumor responses were detected in some patients in whom vaccine-induced T cell infiltrates and neo-epitope-specific killing of autologous tumor cells were found in resected metastases. Since this initial report, therapeutic cancer treatment with personalized mRNA vaccines has received significant attention; several clinical trials listed by the U.S. National Library of Medicine are currently ongoing (clinicaltrials.gov). The inclusion of immune CPIs such as pembrolizumab or zalifrelimab is often a major component of these trials [84].

HCC is a moderately mutated tumor. Recently, Repáraz et al. compared the results of whole-exome sequencing and RNAseq analyses performed on malignant and normal tissues obtained from fourteen HCC patients [107]. A median of 1217 missense somatic single nucleotide variants were identified per patient in malignant tissues; of these, a median of 13 and 5 peptide sequences (neoantigens) per patient were predicted to bind HLA class I and class II molecules, respectively. The immunogenicity of these putative neoantigens was confirmed by demonstrating HLA binding and their ability to elicit human CD4^+^ and CD8^+^ T cell responses in vitro and to activate T cell responses in vaccinated, human transgenic HLA-A*02.01/HLA-DRB1*01 mice. These findings demonstrate the presence of immunogenic neoantigens in HCC tumors that could be incorporated into personalized mRNA-based, anti-tumor vaccines created to target these sequences. Indeed, two preliminary clinical trials (clinicaltrials.gov Identifiers: NCT05192460 and NCT03480152) were undertaken to evaluate the safety, tolerability, and, preliminarily, the efficacy of mRNA-based, neoantigen-specific tumor vaccines in subjects with advanced cancers that include HCC. Notably, NCT03480152 was ultimately terminated due to slow accrual.

#### 4.3.4. Therapeutic Proteins

The development of mRNA-based therapeutics to treat cancer and reshape the tumor microenvironment is receiving increasing attention that extends beyond vaccine production [108]. Systemic administration of LNP-encapsulated mRNAs, which encode therapeutic proteins, provides an excellent approach to treating liver cancer directly. mRNA expression and the production of encoded antibodies in vivo, for example, offer a number of advantages over the injection of recombinant, monoclonal antibodies produced in vitro [109]. Unlike a single bolus of recombinant protein, antibody production post-mRNA administration lasts for several days, dependent upon the stability of the mRNA and normal antibody kinetics [43]. Ideally, the localized production of immunotherapeutic antibodies in the liver, e.g., immune CPIs, stimulates immune responses to HCC while avoiding or reducing the toxicities (i.e., irAEs and CPI-associated autoimmune syndrome) often associated with systemic administration of recombinant immune CPIs themselves.

Similar to immune CPIs, most recombinant cytokines inoculated systemically exhibit a poor safety profile [110,111]. Localized delivery of cytokine-encoding mRNAs formulated in LNPs represents a safer approach to achieving the anti-tumor effects of these cytokines in the liver [108]. IL-12, for example, is a potent mediator of TH1-type immune responses but plagued by a plethora of potentially lethal side effects when inoculated systemically [112]. However, LNP-encapsulated, *IL-12* mRNA administered i.v. in a mouse model of HCC reduced the tumor burden and prolonged survival without eliciting any apparent liver toxicity [113]. Additional analysis indicated that mRNA expression was confined to the tumors and non-malignant regions of the liver. While delivery of mRNA that encodes immune modulators such as cytokines and CPIs is considered a promising strategy to avoid manufacturing, cost, and safety issues, studies that focus on using mRNA/LNP complexes to manipulate these modulators in vivo have not been reported, albeit preclinical studies are ongoing [108].

#### 4.3.5. Adjuvants

LNPs offer an effective means of delivering therapeutic mRNA combined with other factors that improve its efficacy. Co-delivery of an mRNA vaccine and an adjuvant (R848, a novel TLR7/8 agonist), for example, provided concurrent stimulation of both innate and adaptive immune responses with minimal toxic side effects in a syngeneic allograft mouse tumor model. Islam and coworkers reported that mice vaccinated with LNP-encapsulated OVA-expressing mRNA and the chemically modified TLR7/8 agonist C16-R848 exhibited increased tumor-associated antigen presentation, antigen-specific CD8^+^ T cell recruitment, and anti-tumor activity [114].

Similarly, Lee and coworkers reported that incorporating the TLR 2/1 agonist, Pam3CSK4 (PAM3), into LNPs significantly improved the efficacy of an anti-tumor mRNA vaccine [115]. PAM3 synergized with single-stranded mRNA, which triggers innate immunity mediated by TLRs 7 and 8 expressed on endosomal membranes. Mice that were vaccinated with LNP-encapsulated *ovalbumin* (*OVA*) mRNA formulated with PAM3 and subsequently challenged with OVA-expressing mouse lymphoma cells exhibited a marked increase in OVA-specific CD8^+^ T cells, a diminution in tumor size, and an increased rate of survival compared to control groups.

As an alternate approach to adjuvanting and increasing vaccine efficacy, Tse et al. reported that a constitutively active stimulator of interferon genes (STING), which expresses a V155M mutation, acted as a genetic adjuvant when administered in combination with LNP-encapsulated mRNA vaccines [116]. LNP-encapsulated *STING^V155M^* mRNA injected alone into mice induced the rapid production of IFN-α as well as other proinflammatory cytokines, i.e., IL-6, monocyte chemotactic protein-1 (MCP-1) and macrophage inflammatory protein-1β. In a tumor-bearing animal model, mice vaccinated with LNP-encapsulated mRNA-encoded tumor antigen and *STING^V155M^* mRNA exhibited a significant inhibition in tumor growth and increased survival relative to unvaccinated mice or mice vaccinated with mRNA-encoded antigen alone.

### 4.4. Infectious Diseases

Viral hepatitis, inflammation of the liver due to viral infections, is most commonly attributed to the hepatitis A virus, HBV, and HCV in the United States. It is estimated that 1.2 million individuals in the U.S. have chronic hepatitis B and 3.2 million individuals have chronic hepatitis C. Individuals with chronic hepatitis are at an increased risk of developing cirrhosis, fibrosis, and liver cancer.

Treatment of viral hepatitis varies. There has been an extensive effort to prevent HBV infection by vaccination. Albeit if contracted, seven medications (two types of injectable interferons and five oral antivirals) are approved to treat, but not cure, chronic hepatitis B infections. In contrast to HBV, there is no prophylactic vaccine to prevent HCV infections. However, chronic hepatitis C can be readily treated with a drug combination of sofosbuvir and ledipasvir (HCV NS5B and NS5a inhibitors, respectively); a cure rate as high as 96 percent has been reported though treated patients remain susceptible to reinfection. The development of mRNA-based therapeutics for hepatitis viruses A, B, and C offers a potential alternative to preventing and/or treating viral hepatitis.

A number of recent reports have demonstrated the potency and versatility of mRNA vaccine constructs to elicit protective immunity against a wide variety of infectious agents in animal models. mRNA-based vaccines generate potent neutralizing antibody responses in animals immunized with only one or two low doses [117,118,119]. While the results of these animal studies generated a great deal of initial enthusiasm, clinical trials found the immunogenicity elicited by mRNA vaccines to be far more measured in humans than expected based on animal models. With the exception of SARS-CoV-2, no clinical trial undertaken to date has passed the early phase.

SARS-CoV-2 and the coronavirus disease 2019 (COVID-19) pandemic demonstrated the urgent need for technologies that are flexible and able to achieve rapid vaccine development and large-scale production. The FDA granted full approval for the following two mRNA-based SARS-CoV-2 vaccines: both Pfizer-BioNTech (Comirnaty) and Moderna (Spikevax) COVID-19 vaccines are licensed for use in adults and children older than 12 years of age.

#### 4.4.1. Hepatitis B Virus (HBV)

From 5 to 10 percent of adults infected with HBV fail to mount an adequate immune response and subsequently develop chronic hepatitis B. Most HBV-positive children are infected by mother-to-child (vertical) transmission during the perinatal period [120]. Perinatal HBV transmission accounts for 25% of approximately 300M chronic HBV infections worldwide [121]. Importantly, children who are infected very early in life have a significantly higher (90%) chance of developing chronic hepatitis B compared to infections that occur in adults.

Persistent infection is dependent upon the presence of an intrahepatic pool of covalently closed circular DNA (cccDNA) molecules that encode all the gene products required for viral replication [122]. Licensed therapeutics do not impact cccDNA or viral gene expression directly. Engineered nucleases capable of cleaving specific DNA sequences offer a means of permanently incapacitating cccDNA [123]. Recently, Gorsuch et al. described a novel, potentially curative approach to treating chronic hepatitis B using engineered ARCUS nuclease (ARCUS-POL) to cleave the viral genome [124]. Mouse and non-human primate models administered LNP-encapsulated *ARCUS-POL* mRNA systemically exhibited a marked reduction in an episomal AAV vector that contained a portion of the HBV genome that included the ARCUS-POL target site and served as a surrogate for cccDNA. This mRNA-based therapeutic gene editing approach to degrading HBV cccDNA offers a unique treatment for patients with chronic hepatitis.

Alternatively, Chen and coworkers reported that mice in a model of HBV infection [adeno-associated virus (AAV)/HBV] were effectively treated with LNP-encapsulated anti-HBsAg antibody-encoding mRNA [125]. Untreated AAV/HBV mice exhibited persistent levels of serum HBsAg. Treated mice, on the other hand, displayed a marked reduction in HBV seromarkers (i.e., HBV DNA and HBsAg). Passive immunity persisted for ≥30 days. In contrast, mice inoculated with anti-HBsAg antibody produced exogenously demonstrated an initial decrease followed by a rapid recovery of serum HBsAg concentrations by 9 days post-inoculation. Consequently, the authors concluded that the combined effects of the high-affinity anti-HBsAg antibody encoded by the mRNA and the potent adjuvant activity associated with mRNA-LNPs promoted long-term HBsAg seroclearance. The possible contribution of this combined effect to the re-establishment of the immune system in HBV carriers was conjectured.

#### 4.4.2. Cytomegalovirus (CMV)

CMV is related to the viruses that cause chickenpox, herpes, and mononucleosis. It generally causes chronic infections. Although CMV usually remains dormant, it can be reactivated and cause inflammation of the liver and hepatitis in newborns and individuals with weakened immune systems. Indeed, CMV is the principal cause of birth defects in the U.S. and one of the most common infectious causes globally. There is no cure for CMV infections, though antiviral medications, i.e., ganciclovir or valganciclovir administered i.v., can slow viral reproduction. Currently, ModernaTx is conducting a Phase 3 clinical trial in healthy participants to study the safety and efficacy of mRNA-1647, a prophylactic CMV vaccine (clinicaltrials.gov Identifier: NCT05085366).

## 5. Conclusions

The mRNA-based strategies have the potential to provide answers to a variety of modern medical problems. Compared to the transient function of traditional protein drugs synthesized exogenously, mRNA exhibits higher therapeutic efficacy due to continued translation, protein synthesis, and long-lasting expression. Additionally, mRNA offers several advantages over DNA-based approaches. mRNA expression, for example, does not require entrance into the cell nucleus in order to occur, thus eliminating the potential risk of insertional mutagenesis. Moreover, the transient nature of mRNA expression permits precise, controlled dosing in accordance with clinical need. The extensive list of pre-clinical programs dedicated to targeting hereditary diseases that affect pediatric patients is particularly noteworthy. Preliminary evidence suggests that possible treatments of hereditary diseases using mRNA-based technologies could soon enter clinical trials. Naturally, mRNA-based approaches to treating children would require a thorough investigation of safety profiles and therapeutic efficacy relative to both the standard of care and other gene therapy options.

The mRNA therapeutics also offer a promising approach to treating liver cancer and viral hepatitis, especially chronic hepatitis B. While mRNA is capable of inducing strong cellular immune responses, a successful therapeutic vaccine to treat liver cancer has not been reported. Similarly, induction of a robust cellular immune response would benefit efforts to treat chronic hepatitis B patients, though no mRNA-based therapeutic vaccine has been described. Undoubtedly, the biggest obstacle to treating both liver cancer and chronic hepatitis B is generating an effective immune response in the tolerogenic environment characteristic of the liver [126]. In summary, mRNA-based therapeutics promise to be a major factor in strategies to develop drugs to treat a number of liver diseases. 

## Figures and Tables

**Table 1:** Preclinical studies using LNP-encapsulated mRNA constructs to treat rare inherited metabolic liver disorders.

Disorder	Protein Affected	Manifestation	mRNA Construct ^1^	Animal Model	Reference
Fabry disease	Alpha-galactosidase A	Accumulation glyco- sphingolipids	h-*a-Gal A*	*Gla*-deficient, B6;129-Gla^tm1Kul^/J mouse; wild-type NHP	[71]
Type II citrullinemia	Liver-specific mitochondrial aspartate/glutamate transporter (citrin)	Elevated: hepatic citrulline, blood ammonia	h*Citrin*	*Ctrn/mGPD*-double KO mouse	[73]
Classic galactosemia (CG)	Galactose-1 phosphate uridylyltransferase	Elevated: galactose-1 phosphate and plasma galactose	h*GALT* or mGalT	*GalT^−/−^* mouse	[74]
Glycogen storage disease type 1a (GSD1a)	Glucose-6- phosphatase	Hypoglycemia	h*G6PC*	*L-G6PC^−/−^* mouse	[75]
Acute intermittent porphyria (AIP)	Porphobilinogen deaminase	Accumulation porphyrin precursors	h*PBGD*	(*Pbgd*^tm1(neo)UAM^) X (*Pbgd*^tm2(neo)UAM^) mouse; porphyric rabbit; wild-type NHP	[72]
Progressive familial intra-hepatic cholestasis type 3 (PFIC3)	Liver-specific phosphatidylcholine transporter (ABCB4/MDR3)	Cholestasis; progressive biliary fibrosis	h*ABCB4*	*BALB/c Abcb4*^−/−^ mouse	[76]
Arginase deficiency	Arginase 1	Hyperargininemia; guanidino compounds	h*ARG1*	Conditional arginase deficient Arg1^flox/flox^ mouse	[77]
Alpha-1 antitrypsin (AAT) deficiency	SERPINA1 ^2^	Uncontrolled elastolytic activity	h*AAT*	NSG-PiZ mouse ^3^	[78]
Crigler–Najjar syndrome type 1 (CN1)	Uridine-diphosphate- glucuronosyltransferase (UGT1A1)	Unconjugated hyperbilirubinemia	h*UGT1A1*	Gunn-UGT1a1j/BluHsdRrrc rat	[79]

^1^ The construct indicated was encapsulated in LNPs and administered i.v.; h and m prefixes refer to human and mouse sequences, respectively. ^2^ Serine protease inhibitor alpha 1-antitrypsin. ^3^ NOD.Cg-Prkdcscid Il2rgtm1Wjl Tg(SERPINA1*E342K)#Slcw/SzJ.

## Data Availability

Not applicable.
